# Supplementary material for: The genome sequence of the Atlantic white-sided dolphin, Leucopleurus acutus (Gray, 1828) (Artiodactyla: Delphinidae)
Source: Wellcome Open Res. 2026 Jan 5;11:2. [Version 1] doi: 10.12688/wellcomeopenres.25421.1 (PMC13157609; doi:10.12688/wellcomeopenres.25421.1)
Supplement: Peer review history

# Reviewer report: Reviewer response for version 1

**DOI:** 10.21956/wellcomeopenres.28007.r153642

**Published:** 2026-05-09

**Authors:** Luis A. Escobedo-Morales

**Affiliations:** 1 Universidad Nacional Autónoma de México, Mexico City, Mexico

The article is an important resource for genomic research on dolphin species. The methods are suitable for assembling the complete genome, and their description is thorough and clear. The results are well supported. I agree with the previous reviewer's comments; additionally, it would be interesting to include a mention of the previously sequenced resources for the species to provide background and clarify the relevance of the current work.

Are sufficient details of methods and materials provided to allow replication by others?

Yes

Is the rationale for creating the dataset(s) clearly described?

Yes

Are the datasets clearly presented in a useable and accessible format?

Yes

Are the protocols appropriate and is the work technically sound?

Yes

Reviewer Expertise:

Phylogenomics, evolutionary biology

I confirm that I have read this submission and believe that I have an appropriate level of expertise to confirm that it is of an acceptable scientific standard.

# Reviewer report: Reviewer response for version 1

**DOI:** 10.21956/wellcomeopenres.28007.r148770

**Published:** 2026-03-09

**Authors:** Evelien de Greef

**Affiliations:** 1 University of Manitoba, Winnipeg,, Manitoba R3T 2M5, Canada

This article presents the reference assembly of the Atlantic white-sided dolphin, the first for this species. The methods for sequencing, extraction, and assembly are very thorough and clear, and the article includes genome metrics that demonstrate this is a high-quality chromosome-level genome. Data from this work is publicly available and easy to access, and will be a great resource for future work.

 I only have two minor comments. First, I think it would help to elaborate on the rationale for sequencing this genome beyond being a part of the Darwin Tree of Life Project, such as a brief discussion on potential applications of this genome. The other comment I have is to include a little more detail on how the specimen was collected- whether it was stranded, beachcast, hunted, or collected differently.

Are sufficient details of methods and materials provided to allow replication by others?

Yes

Is the rationale for creating the dataset(s) clearly described?

Partly

Are the datasets clearly presented in a useable and accessible format?

Yes

Are the protocols appropriate and is the work technically sound?

Yes

Reviewer Expertise:

Genomics, marine mammal population genetics

I confirm that I have read this submission and believe that I have an appropriate level of expertise to confirm that it is of an acceptable scientific standard.
